# T Cell-Intrinsic and -Extrinsic Contributions of the IFNAR/STAT1-Axis to Thymocyte Survival

**DOI:** 10.1371/journal.pone.0024972

**Published:** 2011-09-20

**Authors:** Hiroshi Moro, Dennis C. Otero, Yoshinari Tanabe, Michael David

**Affiliations:** Division of Biological Sciences and UCSD Cancer Center, University of California San Diego, La Jolla, California, United States of America; Oklahoma Medical Research Foundation, United States of America

## Abstract

STAT1 is an essential part of interferon signaling, and STAT1-deficiency results in heightened susceptibility to infections or autoimmunity in both mice and humans. Here we report that mice lacking the IFNα/β-receptor (IFNAR1) or STAT1 display impaired deletion of autoreactive CD4^+^CD8^+^-T-cells. Strikingly, co-existence of WT T cells restored thymic elimination of self-reactive STAT1-deficient CD4^+^CD8^+^-T cells. Analysis of STAT1-deficient thymocytes further revealed reduced Bim expression, which was restored in the presence of WT T cells. These results indicate that type I interferons and STAT1 play an important role in the survival of MHC class I-restricted T cells in a T cell intrinsic and non-cell intrinsic manner that involves regulation of Bim expression through feedback provided by mature STAT1-competent T cells.

## Introduction

Selection of T lymphocytes expressing antigen receptors that are major histocompatibility complex (MHC)-restricted but tolerant to self-antigens is a fundamental prerequisite for a functional immune system. The relatively small T cell subset of CD4^−^CD8^−^-double negative thymocytes gives rise to the CD4^+^CD8^+^-double positive thymocytes which account for the majority of T cells in the thymus. Subsequently, CD4^+^CD8^+^-thymocytes mature into CD4^+^ or CD8^+^ single positive T cells via a dual selection process. Positive selection occurs if the TCR of immature thymocytes recognizes the MHC on thymic epithelial cells with sufficient affinity to elicit the transduction of survival and differentiation signals. The result is MHC restriction and eventually the development of single positive mature T cells. However, if the TCRs of positively selected T cells subsequently engage in high affinity interactions with the MHC/peptide complex on stromal cells such as dendritic cells and macrophages, these T cells are deleted from the pool via programmed cell death. Thereby, the process of negative selection eliminates self-reactive thymocytes and facilitates tolerance to self-antigens. Collectively, the outcomes of these two selection processes are required to generate a T cell repertoire that is both self-MHC restricted and self-tolerant, a process that appears to be determined by the avidity between the MHC/peptide complex and the TCR [1], [2], [3], [4].

The signal transducer and activator of transcription 1 (STAT1) is a well-characterized component of the type I (IFNα/β) or type II (IFNγ) interferon-induced signaling pathways [5], [6], [7]. The physiological importance of STAT1 in-vivo has been made possible through the generation of STAT1-deficient mice by two independent groups [8], [9]. As anticipated, STAT1-deficient mice failed to respond to either type I or type II interferons, however, comparison of the individual T cell subsets between wild-type (WT) and STAT1−/− 129Sv/Ev mice provided no evidence for significant differences between these two strains [8], [9], [10].

Even though all IFNs utilize STAT1 as a signaling mediator, type I and II IFNs exert opposing effects on the progression of the demyelinating, T cell-mediated autoimmune disease multiple sclerosis (MS) [11]. These juxta-posed effects of type I and II IFNs in MS raise the question as to what role STAT1 plays in this pathological process. To address this issue, we had previously employed mice carrying a transgenic T cell receptor specific for myelin basic protein (TCR^MBP^). Upon T cell activation, TCR^MBP^-transgenic animals develop experimental autoimmune encephalomyelitis (EAE) which serves as a murine model for MS [12], [13]. Analysis of TCR^MBP^STAT1-deficient mice revealed a dramatically increased susceptibility to EAE development, accounted for at least in part by a defect in the development and functionality of CD4^+^CD25^+^ regulatory T cells [10]. However, the severity and frequency of spontaneous EAE development in the absence of STAT1 lead us to hypothesize that STAT1 might also contribute to the events that govern the elimination of autoreactive T cells via negative selection.

Therefore, to explore a possible role for STAT1 in thymic selection, we employed mice carrying a transgenic TCR that recognizes the male-specific HY antigen (designated TCR^HY^) in the context of H-2D^b^, a widely used model system to evaluate thymic selection events [14], [15], [16]. In male animals that harbor the HY antigen, most thymocytes are recognized as self-reactive and deleted from the T cell pool, resulting in the absence of CD4^+^CD8^+^-double positive cells and a severe reduction in thymic cellularity [14], [16].

The findings presented in this study demonstrate that STAT1, as well as type I interferon, is required for the removal of autoreactive T cells in a non-cell intrinsic manner, as TCR^HY^STAT1^−/−^, TCR^HY^IFNAR^−/−^ and TCR^HY^IFNGR^−/−^ animals revealed striking differences in T cell subsets compared to WT littermates. In the model system we employed, the function of STAT1 in T cell apoptosis correlates directly with Bim expression in CD4^+^CD8^+^-double positive cells. As such, our findings support the notion that T cell - T cell interactions, either through direct cell-to-cell contact or via soluble mediators, are essential for adequate T cell development.

## Results

### Impaired elimination of autoreactive T cells in the absence of IFNAR and STAT1

**Figure 1:**
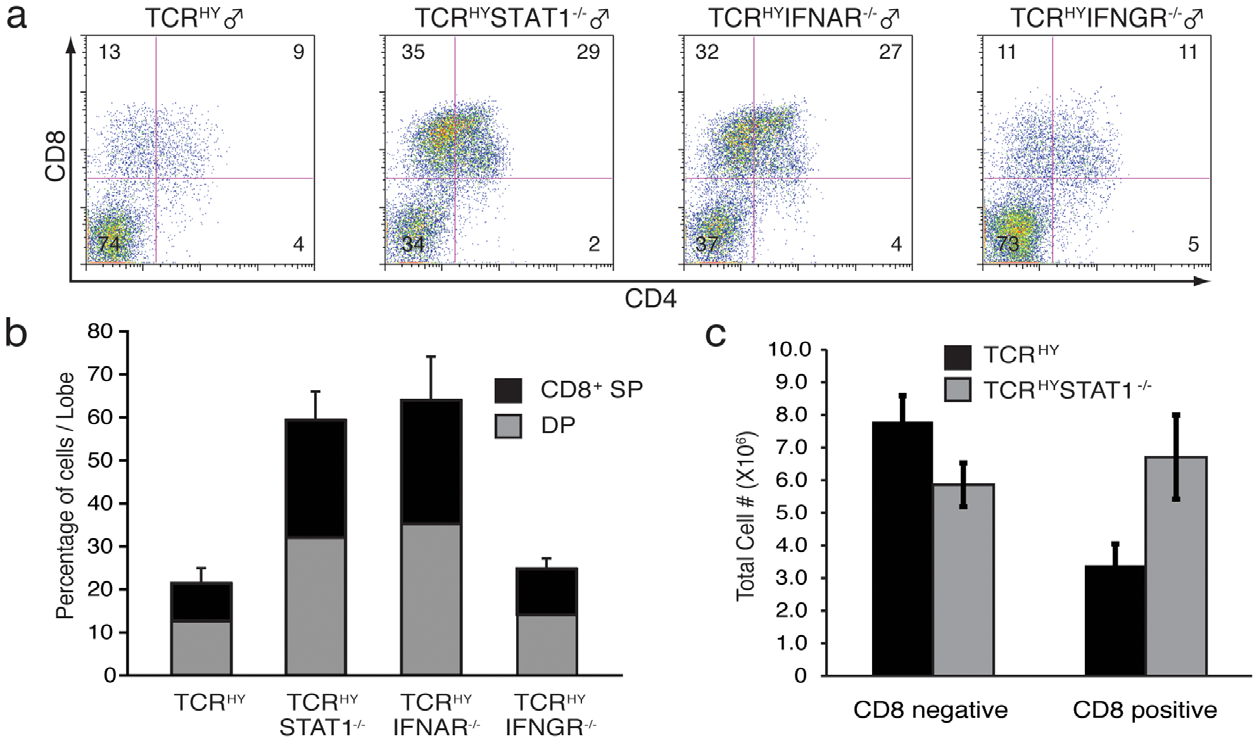
STAT1 and IFNAR are required for elimination of self-reactive TCR^HY^ thymocytes. (**a**) Thymocytes from male TCR^HY^, TCR^HY^STAT1^−/−^, TCR^HY^IFNAR^−/−^, and TCR^HY^IFNGR^−/−^ mice were gated on TCR^HY^ positive cells and analyzed for CD4 and CD8 expressing subpopulations. (**b**) Graph represents percentages of CD8^+^ single-positive (SP) cells (black) and CD4^+^CD8^+^ double-positive (DP) cells (gray) derived from thymi of TCR^HY^ transgenic male mice. Results shown represent averages and standard deviations of three to five experiments. (c) Total numbers of CD8^−^ and CD8^+^TCR^HY^ thymocytes from the indicated mice.

In order to investigate a possible function for STAT1 in thymic selection events, STAT1-deficient mice were crossed with TCR^HY^ transgenic animals yielding TCR^HY^STAT1^−/−^ animals. Thymocytes were isolated from TCR^HY^ and TCR^HY^STAT1^−/−^ transgenic male animals and analyzed by flow cytometry. As previously reported [14], [16], male TCR^HY^ mice almost completely lack CD4^+^CD8^+^-double positive thymocytes in their thymi due to efficient deletion of these autoreactive T cells (Fig. 1) when compared to non-transgenic WT animals or female TCR^HY^ mice (not shown). Strikingly, the absence of STAT1 resulted in a drastic impairment of this elimination process such that a substantial number of CD4^+^CD8^+^-double positive and CD8^+^-single positive thymocytes were detectable in male TCR^HY^STAT1^−/−^ mice (Fig. 1a, 2^nd^ panel). In contrast, deletion of CD4^+^CD8^+^-double positive cells in male TCR^HY^STAT1^+/+^ (Fig. 1a, 1^st^ panel) or TCR^HY^STAT1^+/−^ (not shown) littermates occurred with similar efficiency as observed in TCR^HY^ mice.

As STAT1 functions downstream of the IFNα/β receptor as well as the IFNγ receptor (IFNGR), we decided to explore which if any of these cytokines were accountable for the impaired elimination of the autoreactive T cells. Analysis of the T cell subsets from TCR^HY^IFNAR^−/−^ and TCR^HY^IFNGR^−/−^ animals revealed that only TCR^HY^IFNAR^−/−^ but not TCR^HY^IFNGR^−/−^ mice display a similar defect in the deletion of autoreactive T cells as TCR^HY^STAT1^−/−^ mice (Fig. 1a, 3^rd^ and 4^th^ panel). Thus, the altered distribution of T cell subsets as a consequence of impaired deletion appeared identical in TCR^HY^STAT1^−/−^ and TCR^HY^IFNAR^−/−^ mice, whereas thymic populations in TCR^HY^IFNGR^−/−^ mice resembled those in TCR^HY^ animals (averages of several mice are enumerated in Fig. 1b). In addition, total numbers of CD8^+^ cells were increased in the thymi of TCR^HY^STAT1^−/−^ and TCR^HY^IFNAR^−/−^ mice (Fig. 1c, and data not shown), resulting in the concomitant increased presence of TCR^HY^-positive cells in the spleen, lymph node, and blood (not shown). To exclude the possibility that the observed differences were due to variations in the expression of the antigen receptor, we analyzed the levels of TCR^HY^ in the different strains and found no significant difference between TCR^HY^ and TCR^HY^STAT1^−/−^ mice (Fig. S1), or between the interferon receptor-deficient animals (not shown). Thus, the presence of TCR^HY^CD8^+^ T cells in the thymi of male mice indicates that STAT1^−/−^ thymocytes are failing to be eliminated.

### Impaired deletion of TCR^HY^STAT1^−/−^ thymocytes correlates with the presence of STAT1 in hematopoietic cells

Lee et al. previously reported a cell-intrinsic role for STAT1 in the constitutive expression of MHC class I on lymphocytes [17]. Furthermore, an interferon-independent requirement for STAT1 in MHC class I expression was found specifically in T cells, such that thymocytes and peripheral T cells from STAT1-deficient mice displayed a more pronounced reduction of MHC class I than those derived from IFNAR/IFNGR double-deficient mice [17]. These finding raised the possibility that potentially reduced expression levels of MHC class I on thymic stromal cells is responsible for the attenuated deletion of autoreactive T cells in the absence of STAT1.

We therefore analyzed the surface expression levels of MHC class I in syngeneic WT, STAT1^−/−^, and IFNAR^−/−^ mice. In conformity with Lee et al., we found a dramatic reduction in the surface expression of MHC class I in STAT1^−/−^, but not IFNAR^−/−^ thymic T cells compared to WT cells (Fig. S2, left column panels). In striking contrast to the lymphocytes, however, thymic stromal cells including dendritic cells (DCs) and thymic epithelial cells (TECs) derived from either STAT1^−/−^ and IFNAR^−/−^ mice showed no significant reduction in MHC class I expression levels (Fig. S2, middle and right column panels).

**Figure 2:**
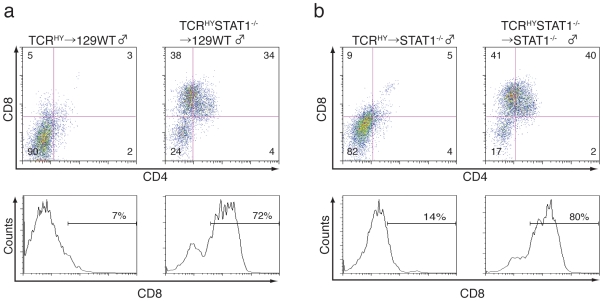
STAT1-deficient T cells fail to undergo deletion in the presence of wild type TEC. BM derived from TCR^HY^ or TCR^HY^STAT1^−/−^ animals was transferred into lethally irradiated male 129WT (**a**) or STAT1^−/−^ mice (**b**). Thymi of recipient mice were analyzed 4 weeks post transfer. Upper panel: TCR^HY^ thymocytes were gated and analyzed for CD4 and CD8 expression, and percentages of T cell subpopulations are indicated. Lower panel: histograms indicate percentages of TCR^HY^CD8^+^ cells. Representatives of at least three experiments are shown.

Due to the fact that thymic stromal cells display normal MHC class I levels we considered that STAT1 maybe required in hematopoietic cells rather than stromal cells for TCR^HY^-mediated deletion. To test this hypothesis, we lethally irradiated male WT mice prior to transfer of bone marrow (BM) derived from TCR^HY^ or TCR^HY^STAT1^−/−^ female mice (Fig. 2a, left panels). Conversely, TCR^HY^-derived BM was also transferred into lethally irradiated non-transgenic STAT1^−/−^ male animals (Fig. 2b, right panels). In such mice, radiation-resistant TECs were derived from the host animal, whereas hematopoietic cells such as T cells, dendritic cells, or macrophages originate from the transferred BM. After four weeks, flow-cytometric analysis of the thymic T cell populations was performed.

As shown in Fig. 2a, adoptive transfer of TCR^HY^ BM into irradiated male WT mice resulted in a similarly efficient deletion of the autoreactive donor T cells as was previously seen in the TCR^HY^ donor animals. However, when TCR^HY^STAT1^−/−^ BM was used to reconstitute irradiated WT animals, autoreactive T cell deletion was diminished to the same extent as was observed in TCR^HY^STAT1^−/−^ mice (Fig. 2a, right panels). Consistent with this observation, adoptive transfer of TCR^HY^ BM into irradiated male STAT1^−/−^ mice yielded animals with intact T cell elimination (Fig. 2b, left panels), whereas the use of TCR^HY^STAT1^−/−^ mice as BM donors led to severely attenuated elimination of the transferred autoreactive T cells in the host animals (Fig. 2b, right panels).

Collectively, these results clearly demonstrate that elimination of TCR^HY^ T cells in male mice is dependent on the STAT1 status of hematopoietic cells, but is independent of the presence of STAT1 in TECs.

**Figure 3:**
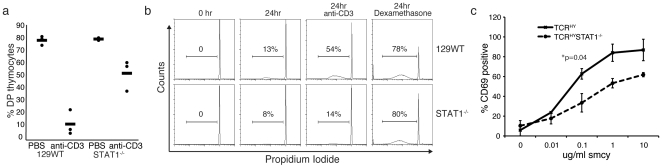
STAT1-deficient thymocytes fail to undergo TCR induced apoptosis. (**a**) Mice were injected i.p. with 20 µg anti-CD3 antibody, and analyzed 48 hours post injection. The percentages of CD4^+^CD8^+^ double-positive (DP) cells in WT and STAT1^−/−^ mice are shown with and without anti-CD3 antibody injection. Results shown represent three mice each. (**b**) Flow-cytometry of PI-stained WT or STAT1^−/−^ thymocytes in culture. Double positive thymic T cells were stimulated with plate bound anti-CD3 for 24 hours, or treated with dexamethasone for 24 hours, and the sub-G_0_G_1_ DNA content of nuclei measured by flow cytometry. Results shown represent averages at least three experiments. **c**) Double positive thymocytes from female TCR^HY^ and TCR^HY^STAT1^−/−^ mice were incubated with indicated concentrations of smcy peptide and analyzed 18 hours later for CD69 expression. Average and standard deviations from 3–4 mice are shown.

To explore whether the observed defects in apoptosis of CD4^+^CD8^+^ double positive thymocytes is linked to the presence of the TCR^HY^, we decided to also evaluate the behavior of this T cell subset in response to different stimulations. The CD4^+^CD8^+^ double positive population of thymocytes is sensitive to crosslinking of the T cell receptor with anti-CD3 antibody and this approach has also been widely used as an alternative strategy to study molecular mechanisms involved in negative selection in vitro as well as in vivo. When 129Sv/Ev WT and STAT1^−/−^ animals were injected with anti-CD3 antibody to induce programmed cell death of thymocytes, STAT1^−/−^ cells showed significantly lower sensitivity to the in-vivo anti-CD3 treatment compared to WT thymocytes (Fig. 3a). To determine if there is an intrinsic defect in TCR-induced cell death of STAT1-deficient thymocytes, double-positive cells from non-transgenic 129Sv/Ev and their STAT1^−/−^ counterparts were stimulated with plate-bound anti-CD3 antibody and apoptosis was measured after 24 hours. Compared to WT thymocytes, double-positive cells derived from STAT1^−/−^-deficient animals displayed significantly reduced apoptosis following T cell receptor crosslinking (Fig. 3b). STAT1^−/−^ T cells are still capable of transducing signals via the TCR as documented by the – albeit somewhat reduced – antigen-induced upregulation of the activation marker CD69 (Fig. 3c). Even though anti-CD3 induced death of double-positive thymocytes in vivo is thought to take place through cytokine secretion by activated CD4^+^ T cells rather than by direct TCR ligation on double-positive thymocytes, the in-vitro experiments using purified double-positive T cells demonstrate that STAT1-deficient thymocytes are indeed less susceptible to programmed cells death via TCR engagement. This difference was specific to TCR-induced apoptosis as STAT1^−/−^ thymocytes were still sensitive to dexamethasone induced cell death in vitro and in vivo (Fig. 3b, and data not shown). Collectively, our results obtained from the adoptive transfer studies and anti-CD3 challenges strongly suggest an intrinsic inability of STAT1^−/−^ thymocytes to undergo deletion triggered by TCR-mediated signals.

### Deletion of DP thymo`cytes requires mature T cell feedback

Our findings suggested that the purge of CD4^+^CD8^+^ TCR^HY^ T cells in male mice is linked to the STAT1 status of hematopoietic cells, but the question remained whether this requirement for STAT1 was intrinsic to the autoreactive T cells. We reasoned that if STAT1 function during selection events is not T cell intrinsic, impaired deletion of TCR^HY^-STAT1^−/−^ CD4^+^CD8^+^ thymocytes might be restored by providing STAT1-expressing hematopoietic cells in trans. We therefore performed RAG-KO complementation assays using mixed BMs in which donor-derived thymocytes represent a mixture of cells that arose from the combined transfer of WT and TCR^HY^-STAT1^−/−^ BMs. Briefly, BM cells derived from the two distinct female donor mice were mixed in a 1∶1 ratio, and injected into lethally irradiated male recipients. To avoid possible contamination of developing donor T cells with residual recipient thymocytes that survived the irradiation, we used male RAG^−/−^ mice as recipients. After 4 weeks, the extent of deletion of TCR^HY^-carrying T cells derived from the transferred BMs was determined by flow cytometry.

**Figure 4:**
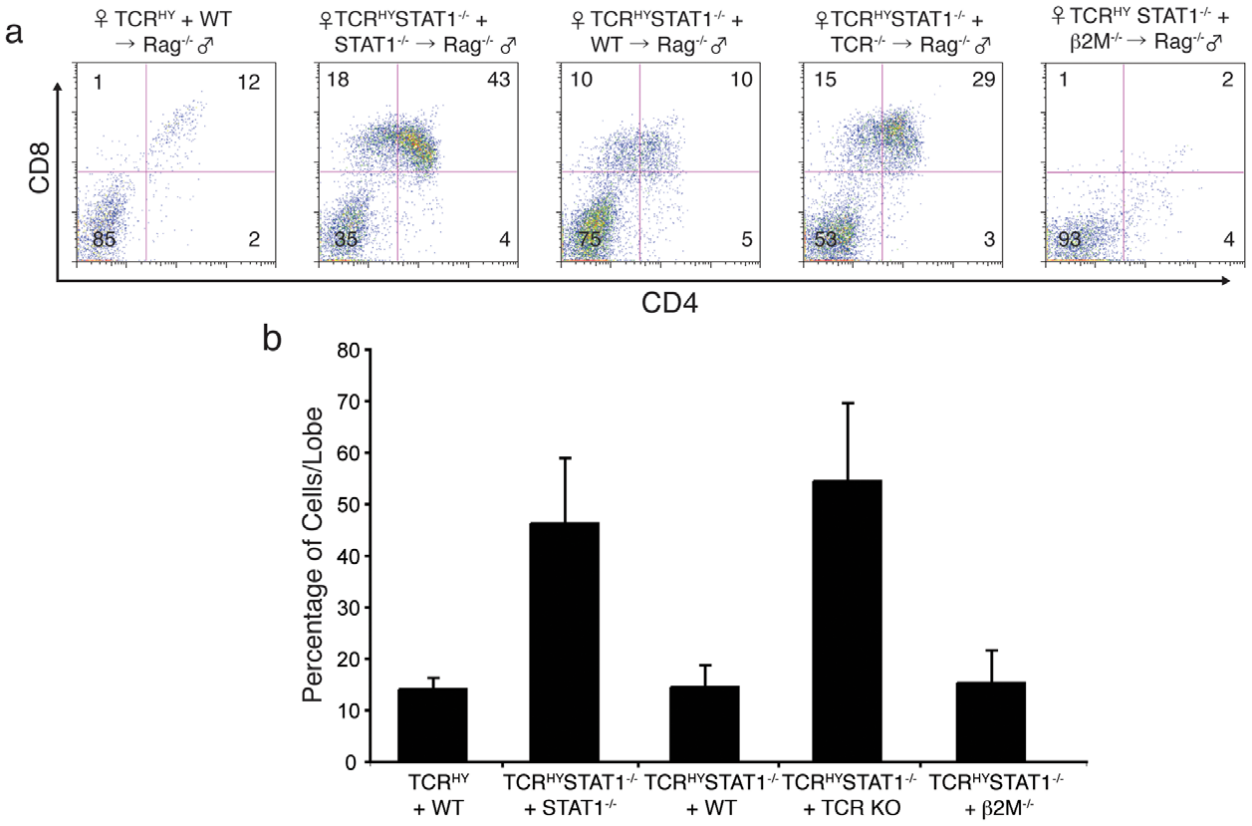
WT T cells promote deletion of autoreactive STAT1-deficient TCR^HY^ thymocytes. (**a**) Flow-cytometry of thymic T cell subsets of chimeric mice. Thymocytes from the indicated BM chimeras were gated on TCR^HY^ cells, and analyzed for CD4- and CD8-expressing subpopulations. The percentages of T cell subpopulations are indicated. (**b**) Graph represents percentages of total TCR^HY^CD8^+^ T cells from the indicated BM chimeras (Average and SD of 4–6 mice each).

As shown in Fig. 4a, when the combined BMs of WT and TCR^HY^ mice were introduced into the irradiated male RAG^−/−^ animals, the elimination of the resulting TCR^HY^ T cells occurred as efficiently as in the original TCR^HY^ mice (left panel). Likewise, transfer of a BM mix consisting of STAT1^−/−^ and TCR^HY^STAT1^−/−^ into the irradiated male RAG^−/−^ animals reproduced the defect of the TCR^HY^-STAT1^−/−^ CD4^+^CD8^+^ thymocytes observed in TCR^HY^STAT1^−/−^ mice (Fig. 4a, 2^nd^ panel). However, when the combined BMs of 129WT and TCR^HY^STAT1^−/−^ were transferred into the irradiated male RAG^−/−^ recipients, the presence of cells derived from the 129WT BM facilitated the adequate elimination of the autoreactive TCR^HY^ T cells (Fig. 4a, 3^rd^ panel). This result indicates that the presence of STAT1-containing hematopoietic cells is a prerequisite for the deletion of autoreactive TCR^HY^STAT1^−/−^ T cells which are intrinsically refractory to elimination.

In an effort to determine which WT BM-derived cell population(s) restored the deletion of TCR^HY^STAT1^−/−^ T cells in these chimeric mice, we also used BM derived from animals deficient in the β and δ chains of the TCR (designated TCR^−/−^). These mice completely lack mature T cells, but otherwise possess a normal hematopoietic cell compartment [18]. Strikingly, when BM derived from the TCR^−/−^ mice instead of 129WT was co-transferred with TCR^HY^STAT1^−/−^ BM, the TCR^−/−^ BM-derived cells failed to restore the eradication of the TCR^HY^STAT1^−/−^ T cells (Fig. 4a, 4^th^ panel). As the only difference in cell types between the 129WT and the TCR^−/−^ donor mice can be found in the presence of STAT1-containing mature T cells in the thymus, it appears that the presence of WT mature T cells is required to facilitate the elimination of developing autoreactive T cells.

The reduced MHC class I expression on thymocytes and the requirement for WT thymocytes in STAT1^−/−^ mice for deletion of self-reactive thymocytes raised the possibility that mature T cells are capable of modulating negative selection of immature thymocytes via MHC class I-mediated interactions. To test this hypothesis we also generated chimeric mice using BMs derived from TCR^HY^STAT1^−/−^ and β2-microglobulin (β2M)^−/−^ animals. TCR^HY^STAT1^−/−^ CD4^+^CD8^+^ thymocytes that co-developed with β2M^−/−^ BM-derived cells were eliminated as efficiently as those that developed in the presence of WT BM (Fig. 4a, 5^th^ panel). These results (summarized in Fig. 4b) demonstrate that the presence of WT T cells is required for removal of developing auto-reactive T cells independent of their MHC class I levels and ability to present self antigen.

### Impaired T cell apoptosis in STAT1^−/−^ mice correlates with reduced Bim expression

**Figure 5:**
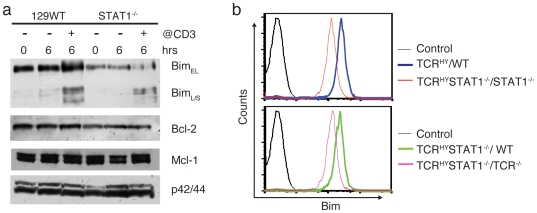
Bim expression correlates with altered elimination of STAT1-deficient thymocytes. (**a**) Immunoblot analysis of Bcl family members in WT and STAT1^−/−^ thymocytes. Cells were isolated from thymi of WT and STAT1^−/−^ mice and cultured in the presence or absence of plate bound anti-CD3 (10 µg/ml). Following 6 hr incubation cells were lysed, subjected to SDS-PAGE and analyzed by immunoblot for the indicated proteins. Lysates were also prepared from freshly isolated thymocytes without further incubation (lanes 1 and 4). Data is representative of at least three independent experiments. (**b**) Flow cytometric analysis of intracellular Bim expression levels in thymocytes from chimeric mice. Chimeric mice were generated as described in Fig. 4, and Bim expression in TCR^HY^-gated thymocytes was determined by intracellular staining using flow-cytometry.

Eradication of self-reactive TCR^HY^STAT1^−/−^ CD4^+^CD8^+^ thymocytes can be restored by the presence of STAT1-containing mature T cells, albeit the resistance of non-transgenic STAT1^−/−^ T cells to anti-CD3 induced apoptosis (Fig. 3) indicates that T cell intrinsic factors are involved in impaired apoptosis in the absence of STAT1. To determine which features are responsible for the impaired elimination of auto-reactive STAT1^−/−^ thymocytes we chose to examine the levels of pro- and anti-apoptotic Bcl-2 family members in STAT1^−/−^ compared to 129WT thymocytes. The pro-apoptotic Bcl-2 family member Bim has been shown to be pivotal to thymocyte negative selection [19] and mice that lack Bim succumb to T cell mediated autoimmune disease [20]. When we analyzed Bim levels of total thymocytes following 0 or 6 hrs in culture with or without anti-CD3 stimulation, we found that Bim levels were strikingly reduced in thymocytes derived from STAT1^−/−^ mice as compared to their wild-type counterparts (Fig. 5a). Splice variants of Bim (Bim_S_ and Bim_ES_) were still induced following TCR stimulation in the absence of STAT1 thymocytes, but maximal Bim levels in STAT1^−/−^ thymocytes still remained well below those observed even in unstimulated WT cells (Fig. 5a, top panel). Bcl-2 levels were slightly lower in STAT1^−/−^ thymocytes (Fig. 5a, 2^nd^ panel from top), whereas no difference was detectable in the levels of the anti-apoptotic family member Mcl-1 (Fig. 5a, 3^rd^ panel from top). As Mcl-1 is considered the direct antagonist to Bim to promote cell survival [21], [22], it seemed reasonable to conclude that the survival of STAT1^−/−^ CD4^+^CD8^+^ thymocytes is promoted by normal Mcl-1 levels in the presence of reduced Bim expression. Consistent with this finding is the observation that STAT1^−/−^ mice develop lymphoproliferative disease as evidenced by significantly enlarged spleens in aged STAT1^−/−^ mice compared to two age-matched WT animals (Fig. S3).

Deletion of TCR^HY^STAT1^−/−^ CD4^+^CD8^+^ thymocytes can be restored by the presence of STAT1-containing mature T cells, one would therefore anticipate that these WT T cells facilitate an increase in Bim expression in the TCR^HY^STAT1^−/−^ CD4^+^CD8^+^ thymocytes. We thus analyzed Bim expression levels by intracellular FACS staining of TCR^HY^STAT1^−/−^ thymocytes obtained from mixed bone marrow chimeras as described in Fig. 4. Corroborating the results acquired from the non-transgenic animals, the expression levels of Bim in TCR^HY^ thymocytes derived from TCR^HY^STAT1^−/−^/STAT1^−/−^ chimeras were significantly lower than in those from TCR^HY^/WT chimeric animals (Fig. 5b, top panel). Consistent with the effects on deletion of TCR^HY^STAT1^−/−^ CD4^+^CD8^+^ thymocytes, the presence of WT, but not of TCR^−/−^ BM-derived cells elevated Bim expression in TCR^HY^STAT1^−/−^ thymocytes (Fig. 5b, bottom panel). In summary, our findings demonstrate an intrinsic inability of auto-reactive STAT1^−/−^ thymocytes to undergo deletion, an event that mandates Bim expression which in turn appears to be controlled by a STAT1-dependent, but cell-extrinsic process.

## Discussion

Deletion occurs when the TCRs of CD4^+^CD8^+^ thymocytes interact with a peptide-MHC complex with high affinity, leading to apoptotic cell death [1], [3], [4], [23]. Our previous work had demonstrated that STAT1-deficiency dramatically increases the incidence of autoimmune disease [10]. In addition to decreased CD4^+^CD25^+^ regulatory T cell function, it seemed plausible that the absence of STAT1 also leads to impaired deletion of auto-reactive T cells in the thymus. Consequently, we decided to investigate whether STAT1 is associated with the central tolerance and to explore this hypothesis, we employed TCR^HY^ transgenic mice deficient in STAT1. The TCR^HY^ is a low affinity receptor compared to other transgenic TCR model systems. The early expression of the TCR^HY^ T cell receptor during thymic development (DN to DP stage) and the universal expression of the HY antigen have provoked questions regarding the immunological relevance of this model. Notwithstanding its limitations, the TCR^HY^ is a widely utilized and effective model in the study of thymic events that control T cell selection processes. Our results revealed that STAT1, in addition to the type I IFN receptor, is indeed required for the removal of autoreactive CD8^+^ cells in the TCR^HY^ model system (Fig. 1a).

IFNs and STAT1 are recognized to play an important role in modulating MHC class I expression [17], and the fact that TCR^HY^-mediated deletion was perturbed in the absence of both STAT1 and IFNAR1 (Fig. 1a) indicated the involvement of type I IFN signals. According to Lee and colleagues, STAT1 is required for the constitutive expression of MHC class I molecules on lymphocytes [17]. Indeed, we observed drastically reduced levels of MHC class I expression on STAT1^−/−^ T cells, however, we did not find a significant decrease in H-2^b^ expression in T cells derived from IFNAR^−/−^ mice (Fig. S2). As deletion was impaired in both TCR^HY^STAT1^−/−^ and TCR^HY^IFNAR^−/−^ mice, where the former had reduced, but the latter displayed normal expression levels of MHC on their thymocytes (Fig. S2), no good correlation exists between the observed defects in deletion of STAT1^−/−^ and IFNAR^−/−^ autoreactive T cells, and lymphocytic H-2^b^ expression. We were also unable to detect any substantial reduction in MHC class I expression levels on thymic stromal cells which are considered the key APCs mediating negative selection. These results imply that the expression levels of MHC class I on thymocytes of TCR^HY^STAT1^−/−^ and TCR^HY^IFNAR^−/−^ mice are unlikely to be (solely) responsible for the defects in T cell selection in these animals.

Most importantly, the coexistence of WT thymocytes restored the impaired deletion of TCR^HY^STAT1^−/−^ thymocytes in male BM chimeras (Fig. 4). However, BM of TCR^−/−^ mice which lack mature T cells failed to support the elimination of the autoreactive TCR^HY^STAT1^−/−^ T cells, strongly suggesting that mature T cells contribute to the efficiency of the selection process. Two groups had independently demonstrated a role for T cell - T cell interactions in the positive selection of CD4^+^ T cells by generating transgenic mice expressing MHC class II on their thymocytes [24]. More recently, it was found that mature single-positive T cells recirculate to the thymus and contribute to the positive selection of thymocytes [25]. Thus, while it appears that positive selection can be induced by MHC expressing T cells, our finding that cell derived from β2M^−/−^ bone marrow can still support deletion of TCR^HY^STAT1^−/−^ thymocytes in mixed BM chimeras suggest that T cell – T cell communication in support of negative selection occurs independent of MHC class I (Fig. 4). It is also important to remember that the donor thymocytes were derived from female mice that do not harbor the HY antigen and can consequently not display the HY peptide antigen in their MHC class I molecules. Therefore, not the MHC class I/antigen complex, but other signals provided by donor-derived T cells via type I IFNs signaling are required for the elimination of TCR^HY^ thymocytes in non-cell intrinsic manner.

The pro-apoptotic factors Bim, Bax and Bak play important roles in T cell development and homeostasis [19], [20], [26]. Bim in particular is important for negative selection through the T cell receptor promoting the removal of self-reactive T cells [19]. Considering that STAT1^−/−^ double positive cells also displayed resistance towards AICD elicited by TCR activation via antibody-mediated CD3 crosslinking (Fig. 3), a possibility existed that STAT1 regulates pro-apoptotic factors. Strikingly, we found that expression of the pro-apoptotic factor Bim was significantly reduced at the protein level in thymocytes from STAT1^−/−^ mice compared to WT mice (Fig. 5a). Bim expression in TCR^HY^STAT1^−/−^ T cells could be restored to WT levels in WT+TCR^HY^STAT1^−/−^ but not in TCR^−/−^+TCR^HY^STAT1^−/−^ chimeric mice (Fig. 5b). Although it has recently been suggested that Bim does not play a role in negative selection, Bim was necessary for elimination of double positive cells in the cortex [27], the stage at which TCR^HY^ T cells are deleted [28]. It is also worthwhile noting that the flow cytometric profiles of male TCR^HY^STAT1^−/−^ thymic cell populations (Fig. 1) closely resemble those of TCR^HY^Bim^−/−^ thymi [19]. In addition, Mcl-1, which is important for T cell development [29] showed no difference in its expression levels between STAT1^−/−^ and WT thymocytes. Thus, STAT1 appears to regulate T cell apoptosis by promoting pro-apoptotic factors rather than by attenuating expression of survival factors.

Taken together, there is mounting evidence that thymocyte-thymocyte interactions play at least an auxiliary role in the deletion of autoreactive T cells. Our current study describes a considerable perturbation of thymic selection in the absence of STAT1 and IFNAR1 that is likely to contribute to the autoimmune disorders in STAT1^−/−^ mice as previously described [8], [9], [10]. It remains to be determined whether this effect is through direct T cell - T cell contact via cell-surface proteins (other than MHC), or by diminishing the production of or response towards T cell-derived soluble factors that affect Bim expression in the developing CD4^+^CD8^+^ thymocytes.

## Materials and Methods

### Animals

STAT1^−/−^ [9], IFNAR^−/−^ [30], IFNGR^−/−^ [31], TCR^−/−^ [18] and TCR^HY^ [16] mice have been described previously. RAG1^−/−^ mice and β2M−/− mice were obtained from Jackson Laboratory (Bar Harbor, Maine). STAT1^−/−^, IFNAR^−/−^ and IFNGR^−/−^ mice were crossed with TCR^HY^ transgenic mice yielding TCR^HY^STAT1^−/−^, TCR^HY^IFNAR^−/−^ and TCR^HY^IFNGR^−/−^, respectively. The expression of TCR^HY^ was verified by FACS analysis. Animals were between 6 and 10 weeks of age at the time of the experiments. All mice used in these experiments were housed in a pathogen-free environment and were bred and cared for in accordance with University of California, San Diego Animal Care Facility regulations. All studies involving animal have been approved by the “The University of California San Diego Institutional Animal Care and Use Committee” (Protocol S02194).

### Flow-cytometric analysis

For immunostaining, single cell suspensions were prepared from thymus, spleen or peripheral lymph nodes. After removal of red blood cells, approximately 10^6^ cells were suspended in FACS buffer (PBS, 1% FCS, 0.02% NaN_3_) and stained for 20 min in the dark on ice. FITC-anti-CD4 (GK1.5), FITC-anti-B220 (RA3-6B2), PE-anti-CD62L (MEL-14), PE-anti-CD69 (H1.2F3), PE/Cy7 or PE-anti-CD8 (53.6.7), APC-anti-CD3 (145-2C11) and biotin-anti-TCR^HY^ (T3.70) were obtained from eBioscience (San Diego, CA). PE-anti-H-2D^b^ (KH95), biotin-anti-CD11c (HL3), biotin-anti-CD44 (IM7), biotin-anti-Rat IgG2a (RG7/1.30) and purified anti-Ep-CAM (G8.8) were purchased from BD Biosciences (San Jose, CA). APC-streptavidin was used as a secondary reagent to detect biotin-labeled mAbs. All samples were analyzed on a FACSCalibur and processed using CellQuest software (BD Biosciences) or Flow Jo (Ashland, OR).

### In vitro and In vivo cell death

Thymi were isolated from 129WT and STAT1^−/−^ mice and single-cell suspensions prepared. Cells were plated at 1×10^6^/ml in 24-well plates coated with 10 µg/ml anti-CD3 (eBiosiences) or 100 nM Dexamethasone (Sigma). Cells were harvested 24 hours following initiation of culture and apoptosis was measured by flow-cytometric analysis of sub-G_0_/G_1_ peaks after propidium iodide (PI) staining in 1 mM Tris (pH 8), 0.1% Triton, 0.1% sodium citrate, 0.1 EDTA, and 50 µg/ml PI. For in vivo studies, mice were injected intraperitoneally with either 20 µg anti-CD3 or Dexamethasone at 1 mg/kg and thymocyte populations were analyzed 48 hours later and compared to PBS treated mice. Double positive thymocytes from female TCR^HY^ and TCR^HY^STAT1^−/−^ mice were incubated with increasing concentrations of smcy (738–746) peptide, Anaspec (Fremont Ca), and analyzed 18 hours later for CD69 expression by flow cytometry.

### Adoptive BM transfers and BM chimeras

For adoptive transfers, recipient mice were subject to 1,000 rad irradiation, followed by tail vein injection of 10^7^ donor-derived BM cells. BM chimeras were generated by isolating 5×10^6^ BM donor cells from two distinct strains, and injecting the combined (10^7^) cells into lethally irradiated (1,000 rad) RAG^−/−^ recipient animals. Recipient mice were housed in a pathogen-free environment with supplementary antibiotic regimen (1 mg/ml Neomycin+0.1 mg/ml Polymyxin-B in water supply), and analyzed 4 weeks post transfer.

### Bim expression

Antibodies used for Western blotting against Bim and p44/42 were from Cell Signaling (Danvers, MA), against Mcl-1 from Rockland (Gilbertsville, PA) and against STAT1 and Bcl-2 from BD Bioscience. Intracellular Bim was detected with antibodies from Cell Signaling using the Fixation and Permeabilization kit from eBioscience.

## Supporting Information

Figure S1 **TCR expression on TCR^HY^ and TCR^HY^STAT1^−/−^ thymocytes.** Total thymocytes from TCR^HY^ and TCR^HY^STAT1^−/−^ male mice were stained with monoclonal antibody T3.70 recognizing the Vαβ chains of the TCR^HY^ transgenic TCR. Dashed and solid lines represent TCR^HY^ and TCR^HY^STAT1^−/−^ thymocytes, respectively.
(TIF)

Figure S2 **MHC class I expression in WT and STAT1−/− thymi.** Flow cytometric analysis of thymocytes derived from WT, STAT1^−/−^ and IFNAR^−/−^ mice stained with MHC class I H-2^b^-specific antibodies. Histograms are derived from CD3^+^-gated T cells, CD11c^+^-gated dendritic cells or B220^−^Ep-CAM^+^-gated thymic epithelial cells, respectively. Dotted lines in the middle and lower panels represent MHC class I expression in WT cells, and the mean fluorescence intensity (MFI) is indicated. Representatives of at least three independent experiments are shown.
(TIF)

Figure S3 **Lymphoproliferative disease in STAT1−/− mice.** **a**) Spleens from four ∼36 week old STAT1^−/−^ and two age matched WT mice are shown for comparison. **b**) Flow-cytometric analysis of splenic T cells from WT and STAT1^−/−^ mice: Splenocytes from the indicated mice were gated on CD3^+^ cells, and analyzed for CD62L and CD44-expressing subpopulations.
(TIF)
