# Supplementary figures and images for: T Cell-Intrinsic and -Extrinsic Contributions of the IFNAR/STAT1-Axis to Thymocyte Survival
Source: PLoS One. 2011 Sep 20;6(9):e24972. doi: 10.1371/journal.pone.0024972 (PMC3176796; doi:10.1371/journal.pone.0024972)

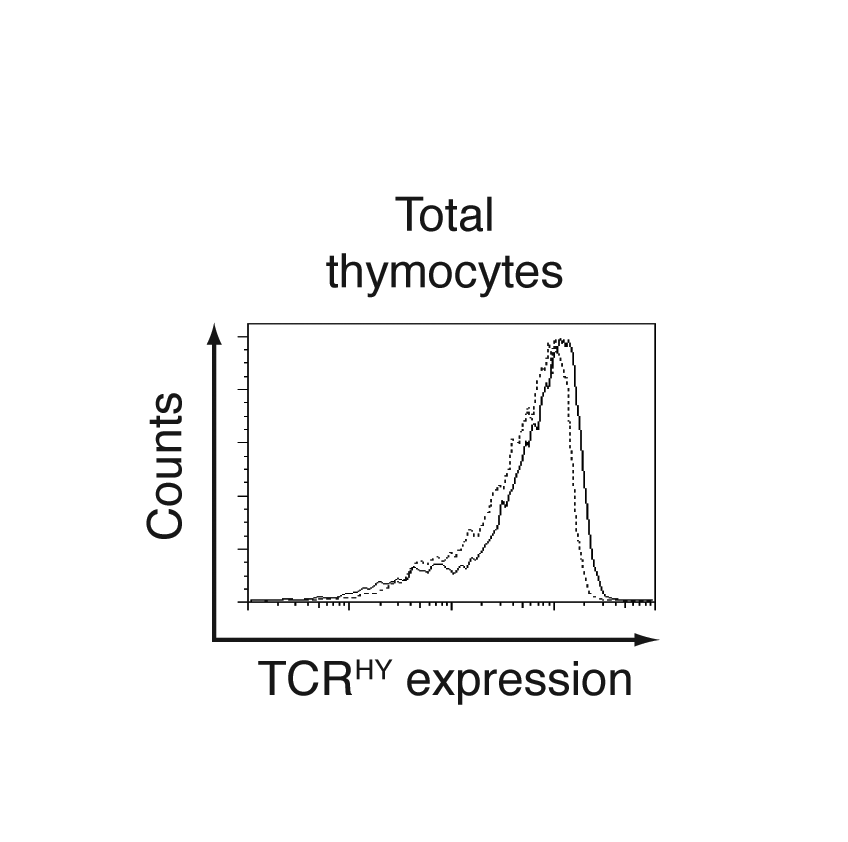

Supplement: Figure S1 — TCR expression on TCRHY and TCRHYSTAT1−/− thymocytes. Total thymocytes from TCRHY and TCRHYSTAT1−/− male mice were stained with monoclonal antibody T3.70 recognizing the Vαβ chains of the TCRHY transgenic TCR. Dashed and solid lines represent TCRHY and TCRHYSTAT1−/− thymocytes, respectively. (TIF) [file pone.0024972.s001.tif]

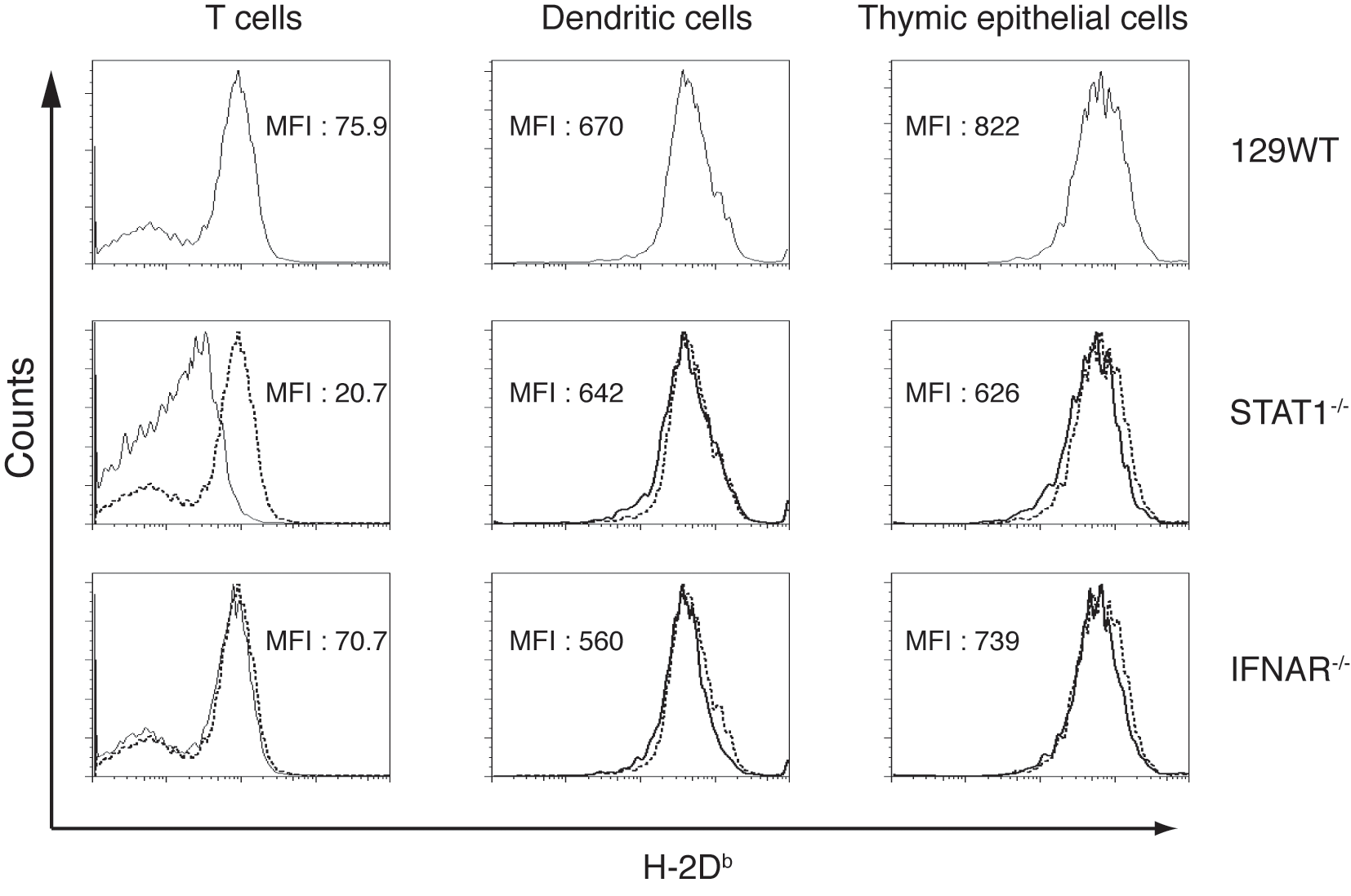

Supplement: Figure S2 — MHC class I expression in WT and STAT1−/− thymi. Flow cytometric analysis of thymocytes derived from WT, STAT1−/− and IFNAR−/− mice stained with MHC class I H-2b-specific antibodies. Histograms are derived from CD3+-gated T cells, CD11c+-gated dendritic cells or B220−Ep-CAM+-gated thymic epithelial cells, respectively. Dotted lines in the middle and lower panels represent MHC class I expression in WT cells, and the mean fluorescence intensity (MFI) is indicated. Representatives of at least three independent experiments are shown. (TIF) [file pone.0024972.s002.tif]

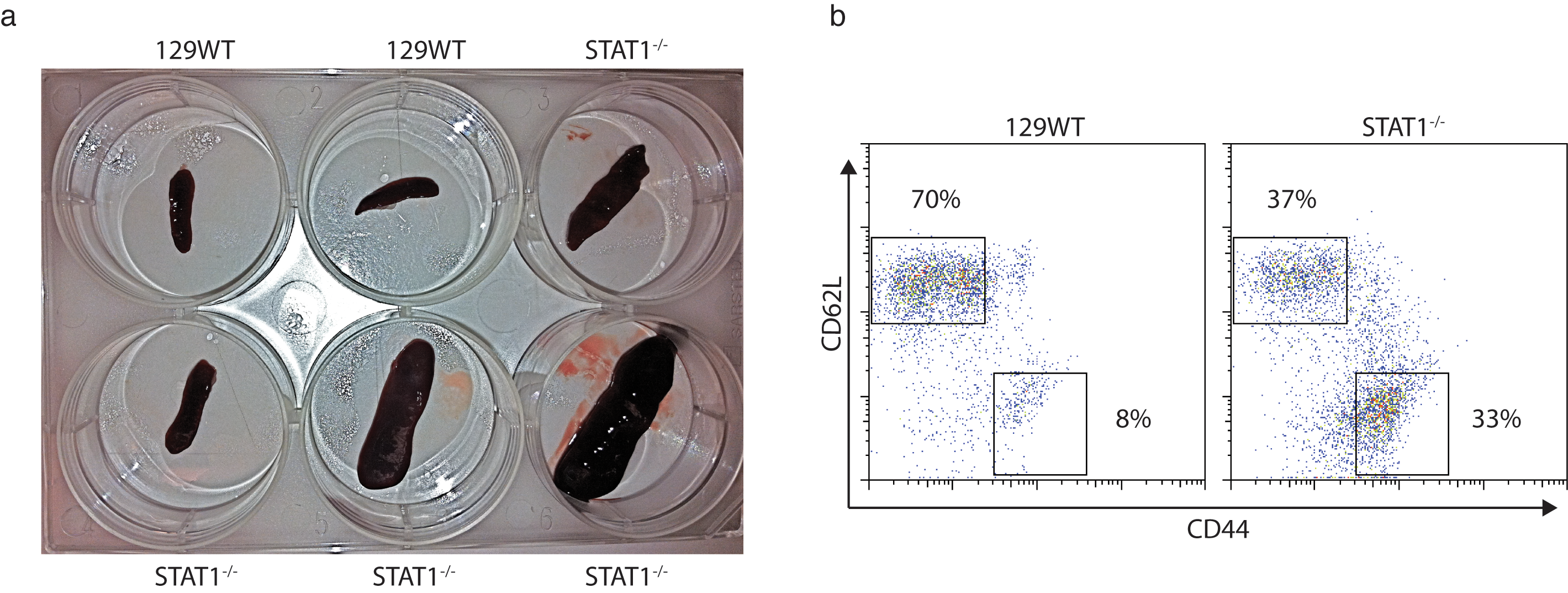

Supplement: Figure S3 — Lymphoproliferative disease in STAT1−/− mice. a) Spleens from four ∼36 week old STAT1−/− and two age matched WT mice are shown for comparison. b) Flow-cytometric analysis of splenic T cells from WT and STAT1−/− mice: Splenocytes from the indicated mice were gated on CD3+ cells, and analyzed for CD62L and CD44-expressing subpopulations. (TIF) [file pone.0024972.s003.tif]
